# Radiologic Evaluation of Small Renal Masses (II): Posttreatment Management

**DOI:** 10.1155/2008/918050

**Published:** 2008-09-25

**Authors:** J. Santos, C. Deltoro, M. I. Martín, A. Marhuenda

**Affiliations:** Departamento de Radiología, Instituto Valenciano de Oncología, C/ Profesor Beltrán Báguena 8, 46009 Valencia, Spain

## Abstract

The increase in the detection of small renal masses (SRMs) and their best knowledge leads to a change in the therapeutic management of these lesions. The use of a less aggressive surgical technique or even an expectant attitude is the current tendency, in order to preserve as much renal function as possible. Imaging techniques are essential in the followup of these lesions. It allows us to know the postsurgical changes and possible complications due to treatment and the presence of local recurrence and metastases. Furthermore, a close radiological followup of SRM related to ablative treatments is mandatory. The purpose of this article is to reveal the imaging features of complications due to surgical or ablative treatments, local recurrence and metastasis, as well as their followup.

## 1. IMAGING FOLLOWUP OF SRM

Several authors have reported that small incidentally detected tumors are associated with better survival outcomes. The 5-year disease-free survival rate for incidental renal tumors of <4 cm treated with radical or partial nephrectomy is 95%–100%. There is a clear increased rate of metastases in patients found to have renal cell carcinoma (RCC) >3 cm in maximum dimension at autopsy compared to those with RCCs of < or =3 cm [1, 2].

Silverman et al. [3] have established the indications for percutaneous biopsy of renal masses in patients with a renal mass and known extrarenal primary malignancy, imaging findings that suggest unresectable renal cancer, surgical comorbidity, those that may have been caused by an infection. Emerging indications are patients with a small (<3 cm) hyperattenuating, homogeneusly enhancing renal mass, those with a renal mass considered for percutaneus ablation and patients with an indeterminate cystic renal mass.

After surgical treatment, radical nephrectomy (RN) or partial nephrectomy (PN), about 20%–30% of patients with localized renal tumors relapse [4]. The recurrences occur three years after surgery, with a median time to relapse being 1 to 2 years. In multifocal renal cortical tumors, local recurrences rates following elective partial nephectomy are from 0% to 10% with a risk of local recurrence for tumors of 4 cm or less [5]. However, late tumor recurrences can occur many years after treatment. The lung is the most vulnerable site for distant recurrence (50%–60% of patients) [6]. Other sites of recurrence are bone, surgical site, brain, liver, and the contralateral kidney.

There are multiple prognostic factors to predict recurrence after surgery. A postoperative prognostic nomogram has been published predicting recurrence for patients with conventional clear cell renal cell carcinoma [7], and it can be useful for patient counselling, clinical trial, and effective patient followup strategies.

Greatest tumor diameter, T stage, stage group, and nuclear grade are important factors in determining the likelihood of recurrence. At the present time, active surveillance of small renal masses is an experimental approach, but represents an attractive option for elderly patients and those with significant comorbidity.

Bilateral multifocal renal tumors are present in approximately 5% of patients with sporadic renal tumors [8]. Conventional clear cell carcinoma is the most common histologic subtype, followed by papillary carcinoma [5]. Most of them can be synchronous but asynchronous lesions may occur many years after the initial nephrectomy, and that is why a long-term followup. must be maintained.

In imaging followup evaluation of kidney cancer, CT is the modality of choice for detection of local recurrence and distant metastases. In patients with compromised renal function or with contraindications to iodinated contrast, gadolinium-enhanced MR imaging of the abdomen and pelvis may be used. Also a chest radiograph or chest CT study can be performed for surveillance of pulmonary metastasis.

Renal cysts are common benign lesions and are often an incidental finding during abdominal CT, (see the appendix) [9]. If they are of fluid attenuation, lack internal architecture, have thin walls, and show no evidence of enhancement after IV contrast administration, they can be easily dismissed as benign. However, the appearance of moderately complex or mild renal cyst varies and can cause difficulties in diagnosis and management. The Bosniak classification or renal cysts has proven to be a useful tool in helping to evaluate these lesions and decide clinical management [10]. In 1993, Bosniak revised the original classification system [11] to include a subset of category II lesions, category IIF lesions (“F” for followup).

CT studies are an effective way of managing patients with moderately complex cystic lesions of the kidney (Bosniak category IIF) because the absence of change supports benignity and progression indicates neoplasm. Alternatively, MRI may prove helpful in the characterization of these lesions and may possibly avoid the need for followup examinations in these cases [12]. In these lesions considered to be category IIF, the followup examinations are necessary to prove stability and, therefore, benignity. The first followup examination is recommended 6 months after the initial examination. If the lesion is unchanged, additional followup examinations should be performed at yearly intervals for at least 5 years, although the optimal followup period has not been determined. However, in younger patients, a longer followup period may be necessary.

**Figure 1:**
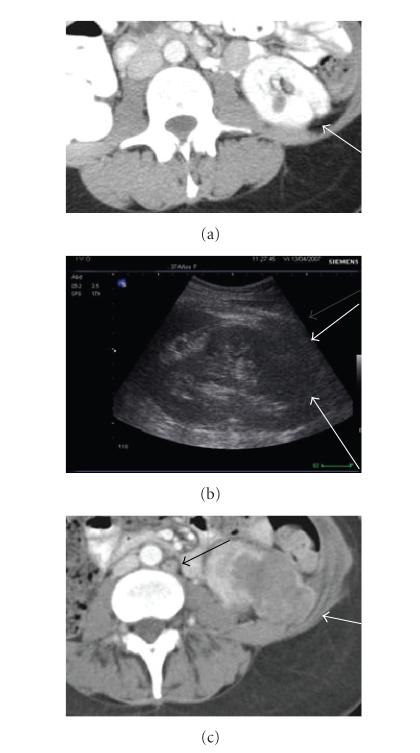
37-year old woman with von Hipple Lindau disease. Radical right nefrectomy and partial tumorectomy in left kidney. (a) Axial contrast-enhanced CT scan shows scar in the lower pole in the left kidney (*arrow*), without any mass. Note the absence of right kidney. (b) One year later sagittal US scan with a large mass less echogenic than renal sinus fat, involving the lower pole parenchyma (arrows). (c) Axial contrast-enhanced CT (nephrographic phase) scan obtained at the same time shows the mass that has grown with perinephric extension (white arrow). It was a renal adenocarcinoma. Note of the presence of paraaorthic lymph node (black arrow).

45% of the patients with von Hippel-Lindau disease will have a renal adenocarcinoma, often (80%) multifocal or bilateral. Treatment must be as conservative as possible because of the multifocality and its usual low grade. The risk of recidive is very high: 30% at 5 years, 80% at 10 years, therefore, they must be followed up strictly and regularly (Figure 1) [13].

## 2. IMAGING OF COMPLICATIONS OF PARTIAL NEPHRECTOMY

The standard treatment for renal cell carcinoma was, for many years, radical nephrectomy, but over the past 10 years, there has been a trend toward the use of nephron-sparing surgery to treat renal cell carcinoma. The results of numerous studies have demonstrated equivalent cancer survival rates for patients who underwent radical nephrectomy and those who underwent partial nephrectomy for small renal neoplasms [14–16].

The procedure can be performed by using open or laparoscopic techniques. However, partial nephrectomy with laparoscopic techniques is a more complex operation than the traditional radical nephrectomy and higher complication rates have been reported [17].

It is important to know normal findings and imaging features of postsurgical complications after partial nephrectomy, for appropriate postoperative management.

### 2.1. Postoperative appearance

**Figure 2:**
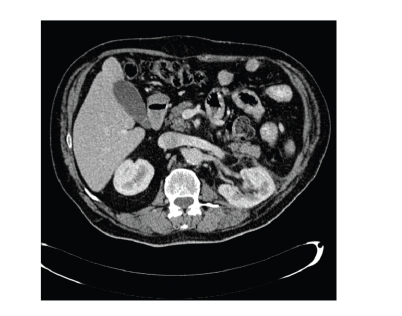
Postoperative findings after laparoscopic left partial nephrectomy, image shows a posterior location of the left kidney, which abuts the posterior abdominal wall.

The appearance of the postoperative kidney depends on the size and location of the resected tumor. After partial nephrectomy for a small peripheral tumor, a wedge-shaped defect in the renal parenchyma is typically visible at CT and MR imaging. The postoperative kidney usually has a more posterior location and abuts to the posterior abdominal wall (Figure 2). Perinephric fat maybe packed into the surgical bed to help achieve haemostasis. This material may be mistaken for a fatty mass such as an AML.

To help control intraoperative bleeding, biologically absorbable haemostatic agents also may be used. Such materials may contain bubbles or air pockets that may resemble a focal abscess. The possible presence of a haemostatic agent should be considered if a linear arrangement of air bubbles is noted or if air bubbles maintain the same position on subsequent images. In most cases, the air in a haemostatic agent is rapidly reabsorbed during the first postsurgical week. However, in some cases, air bubbles can be identified on images even 1 month after surgery. The presence of an abscess should be suspected if a localized fluid collection that has an enhanced rim and contains gas bubbles or a gas-fluid level is seen. In addition, decreased intensity of the nephrogram because of edema in the surrounding renal parenchyma supports the diagnosis of an abscess. Of course it is necessary to consider the imaging findings in combination with the patient's clinical history and symptoms [18–20].

The biologically absorbable haemostatic agents may also mimic a pseudotumor that can lead to confusion. Several cases have been reported on literature after nephron-sparing surgery using gelatine bio absorbable sponge. They were seen as solid masses, with regular borders and enhancement after injection of intravenous contrast agent, due to the presence on granulomatous tissue surrounding the haemostatic material. In all the cases there was a complete resolution of such lesions in an average time of thirteen months [21, 22].

### 2.2. Complications

Complications seen on partial nephrectomy can be divided into vascular complications, complications in the collecting system, infection, recurrent tumor and complications due to technical factors [20].

#### 2.2.1. Vascular complications

**Figure 3:**
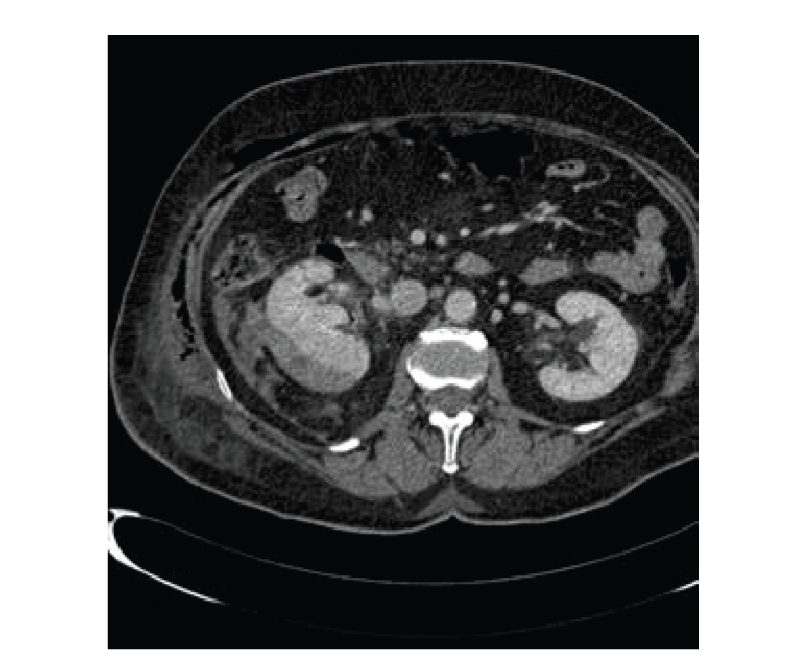
Hematoma after open right partial nephrectomy. Mass with attenuation of 60HU that extends from postoperative bed to the perinephric space.

During partial nephrectomy, the renal hiliar vessels must be temporarily clamped to ensure a bloodless surgical field; however, clamping may injure the arterial intima and lead to thrombosis. If that complication is not recognized at the time of surgery or in the immediate postoperative period, renal infarction and atrophy will occur. Complications related to injury of the intrarenal arteries in the surgical bed may also occur. A hematoma may result if the suturing of transected blood vessels is inadequate (Figure 3). A pseudoaneurysm may result from injury to an intrarenal artery at the surgical site or to the main renal artery or one of its major branches [23–25].

#### 2.2.2. Complications in the collecting system

When calyceal entry is necessary, it would have to be repaired in order to avoid urinary leakage. If the repair is not watertight, a urine leak may occur into the surgical bed. Such leakage may have the appearance of a simple fluid collection in the perirenal space [26], or it may have a more heterogeneous appearance if it contains blood products. This complication can be diagnosed on the basis of contrast-enhanced CT and MR images acquired during the excretory phase, with the observation of contrast material leakage from the collecting system into the surgical bed. In most cases, the fluid collection resolves either spontaneously or after placement of a ureteral stent or nephrostomy catheter. Less commonly, urinary leakage persists and an urinoma forms [20].

#### 2.2.3. Infection

A fluid collection in the surgical bed may become infected, and an abscess may develop. With imaging techniques alone, it may be difficult to differentiate an infected fluid collection from an uninfected one. Moreover, as mentioned before, the presence of air bubbles in a bioabsorbable haemostatic agent may further complicate the interpretation of imaging studies. However, patients with a postoperative abscess are likely to manifest clinical symptoms and signs suggestive of infection; in such cases, a needle aspiration is performed for laboratory analysis, followed by drainage if necessary. In addition, patients who have undergone a partial nephrectomy may present with symptoms of pyelonephritis, which may appear as a striated or heterogeneous nephrogram and may be difficult to differentiate from renal infarction on images alone [20].

#### 2.2.4. Complications due to technical factors

During partial nephrectomy, the liver or spleen may be inadvertently lacerated or contused by surgical instruments used to keep adjacent organs away from the surgical field. Such injuries may be detected with CT and MR imaging. In addition, hernias may occur at the incision site and may contain portions of the bowel or other abdominal organs [26].

## 3. IMAGING OF LOCAL RECURRENCE

**Figure 4:**
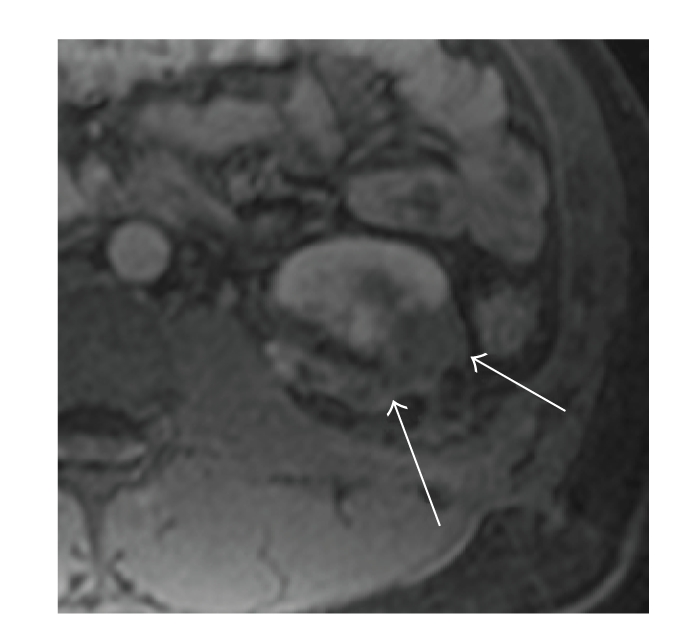
Fibrosis. Axial post contrast T1 with fat saturation shows a hipointense lesion on the lateral aspect of the left kidney that does not enhance with gadolinium, revealing post surgical changes.

**Figure 5:**
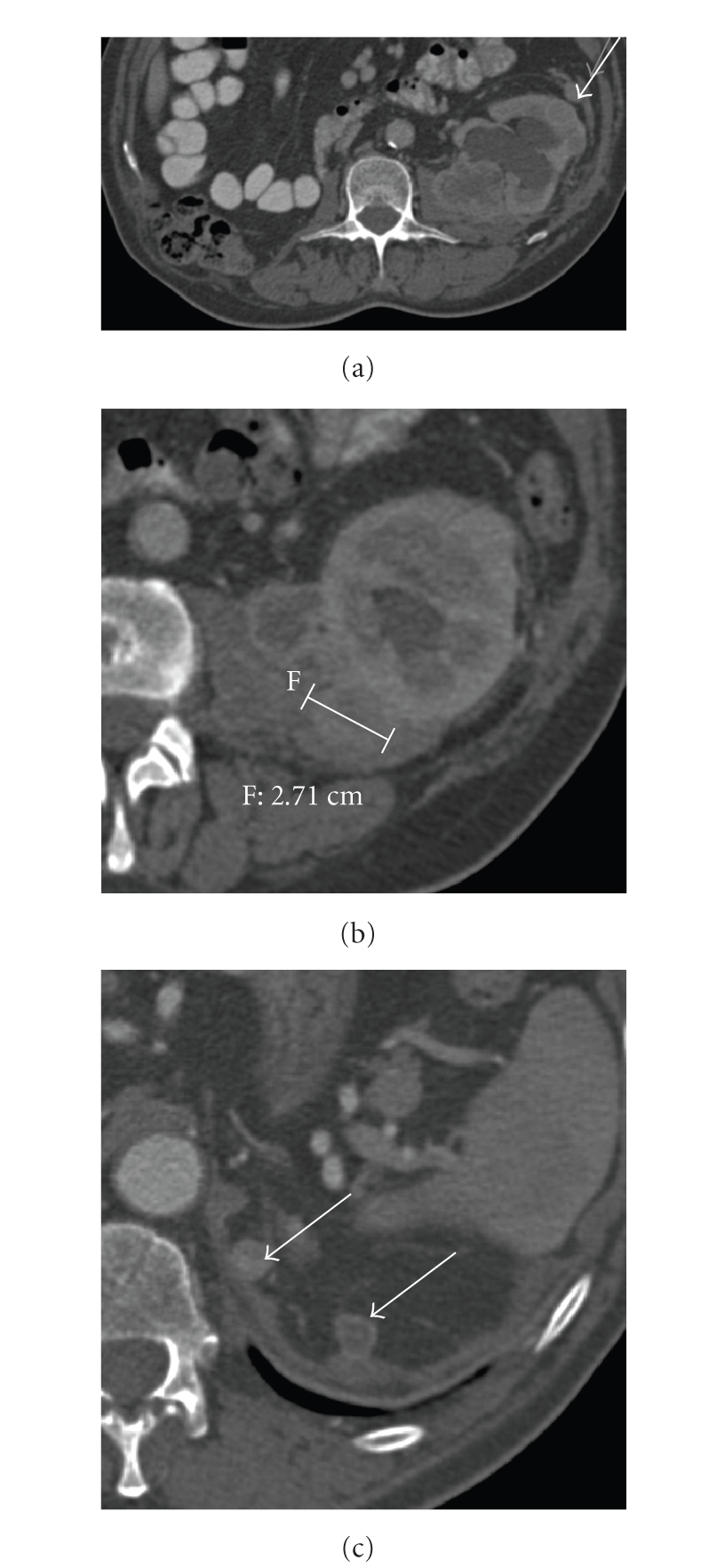
Local recurrence. (a) Axial contrast enhanced CT scan showing the postsurgical changes on the right lumbar fosse with removal of the kidney and migration of the right colon to the nephrectomy fosse. Note the second small renal lesion on the anterior pole of the left kidney, probably due to multifocality that was not advertised on the previous studies of the patient (arrow). (b) A soft tissue mass on the medial aspect of the left kidney at the site of the previous enucleation resection with enhancing nodules around the kidney and adjacent to the psoas. (c) Enhanced nodules adjacent to the diaphragm muscle (arrows).

The most important risk factor for recurrence is the surgical stage of renal cell carcinoma at the time of diagnosis, being for large tumors a bigger incidence than for small ones. However, size is not of prognostic value if capsule is not invaded (13). Patients with positive nodes at surgery relapse sooner, and factors like a high Fuhrman grade on histopathology, and collecting duct carcinoma spindled (sarcomatoid) tumor architecture also adversely influence prognosis [27]. Recurrence must be differentiated from postsurgical fibrosis (Figure 4) and multifocality within the kidney, probably more often seen since small renal tumors are managed with conservative surgical techniques (Figure 5) [13].

The possibility of local recurrence in the remaining kidney is the main limitation of nephron-sparing surgery in patients with renal-cell carcinoma. Local recurrence occurs in about 5% of patients, and has been related to cancer multifocality, incomplete resection of the primary tumor, positive surgical margins, or regional lymph node metastasis. Some authors reported that the type of surgical intervention (enucleation, enucleoresection and resection) does not affect the frequency of tumor local recurrence [28].

Recurrence usually occurs within the first five years after surgery, but late recurrence has been related to renal cancer and long-term followup after a nephrectomy is mandatory for patients with perinephric invasion of a renal cell carcinoma due to the risk of renal fosse recurrence [29]. Followup of these patients is usually made by CT but also MRI for selected cases, as mentioned in the previous article and, in both techniques, arterial phase scanning is essential for maximizing lesion conspicuity, followed by a portal venous phase. Owing to the increased risk of these patients for additional renal primary carcinomas, the renal fosse and remaining kidney must be carefully evaluated looking for a recurrence.

### 3.1. Local recurrence in renal fosse after nefrectomy

Recurrent cancer after nephron-sparing surgery can be suggested when an enhancing nodule develops in the wedge-shaped partial nephrectomy defect. After radical nephrectomy at imaging, retroperitoneal anatomy is significantly altered after surgical removal of the kidney. Small bowel, spleen, pancreas, and colon may migrate into the nephrectomy fosse [30] (Figure 5(a)).

At partial nephrectomy if an adequate margin is not obtained and surgical excision is incomplete, the growth of any remaining neoplastic cells at the resection site over time may result in tumor recurrence in the surgical bed. Even if a tumor is completely excised, it may recur if tumor cells are spilled into the surgical field at the time of resection.

Alternatively, in a patient with multiple foci of disease, an apparent tumor recurrence may actually be an additional preexistent renal cell carcinoma that either was not depicted at preoperative imaging studies or was not identified intraoperatively [23]. The surgical field of view during laparoscopic partial nephrectomy is limited, and the surgeon can see only a small portion of the kidney. This limitation may lead to a failure to identify a specific small renal tumor if there is more than one small lesion in the vicinity. Unless previous imaging studies are carefully reviewed, the latter then might be misidentified as a recurrent lesion.

The radiologic presentation of a recurrent renal carcinoma after surgery appears as an enhancingmass in the surgical site. The recurrence often involves the quadratus lumborum and psoas muscles andcan displace or invade nearby structures, even the spine. The cephalic extent may reach the adrenal bed or may involve the ipsilateral adrenal gland if the latter was spared at the time of nephrectomy [31].

Moreover, as mentioned earlier, bioabsorbable haemostatic agents may be seen as pseudotumor, so, a close followup examination is required to see the evolution.

### 3.2. Local recurrence and residual disease after thermal ablation

Early detection of a recurrence following initial treatment is mandatory for any surveillance protocol, and it is essential to review the preablation and ablation images for a good interpretation of followup images. Imaging must be carefully evaluated to determine the initial tumor size, tumor location, and electrode placement in an effort to predictare as that are likely to demonstrate recurrence. Eccentric electrodeplacement within a mass is likely to result in residual disease at the tumor margin farthest from the ablation device tip. Occasionally, a new tumor focus may develop.

As with local recurrence, residual tumor is suggested when enhancing nodules or crescents areas noted in the vicinity of the treated tumor on contrast enhanced CT or MR images. Furthermore, gadolinium-enhanced fat-suppressed T1-weighted subtraction MR images are helpful in demonstrating subtle areas of enhancement by eliminating the high signal intensity often present within the tumor on unsubtracted images. Because the ablation zone following RF ablation typically has low signal intensity on T2-weighted MR images, a new or enlarging focus of hyperintensity on these images may also be a sign of viable tumor.

Ablated tumors remain stable in size or involute over time on followup images. Therefore, an increase in tumor size after the acute postablation changes have resolved should raise concern for tumor recurrence, as well as within the renal vein and inferior vena cava, even in the electrode insertion site [30].

## 4. FOLLOWUP IMAGING AFTER RADIOFREQUENCY ABLATION OF SRM

There has been a clear increase in the incidence of RCC during the past 10 years, as a result of an increased rate of incidental detection of renal neoplasm. It has been reported that radiofrequency ablation can completely destroy renal cancers, while transmitting minimal collateral damage to surrounding renal parenchyma [32].

Radiofrequency ablation is a safe effective treatment for small renal-cell carcinoma (RCC) in selected patients who are not good operative candidates. Small size and noncentral location are favorable tumor characteristics (large tumors can sometimes be successfully treated but could result in an increased risk of residual RCC). After ablation, computed tomography or magnetic resonance imaging is used to confirm complete eradication or the presence of residual unablated tumor. When the appearance of the ablated tumor deviates from expected findings, percutaneous biopsy is necessary to further evaluate the ablation zone [33].

### 4.1. Imaging followup

All patients must undergo contrast-enhanced imaging (MRI or CT) before radiofrequency ablation as a baseline comparison for subsequent imaging after ablation (initial tumor control).

#### 4.1.1. CT imaging

CT scan of the kidney must be obtained immediately after the ablation session to assess tumor destruction. Normal tissue shows enhancement, with no enhancement in treated area, which encompasses tumor. Small gas bubbles are seen in area of treatment, this is an expected finding resulting from tissue boiling during ablation [34]. After ablation an initial CT scan, imaging followup without and with contrast agent must be performed after 1 month, 3 months, and 6 months and subsequent followup will depend on the clinical condition of the patient and the comorbid conditions, generally at 6 to 12 month interval. Enhancement of any portion of the tumor must be considered residual viable tumor, and the absence of enhancement as no evidence of disease (complete necrosis and thus completely ablated tumor). Images must also be reviewed for the presence of any new metastatic disease or new renal cell carcinomas [34, 35].

#### 4.1.2. MR imaging

A considerable number of patients of eligible patients cannot receive contrast agents that contain iodine because of preexisting impaired renal function or severe contrast material allergies. These patients are usually referred for contrast enhanced magnetic resonance (MR) imaging of the kidney. As in CT imaging, followup MR imaging must be performed in all patients immediately after the completion of the RF ablation. At T2-weighted fast SE images performed, the ablation zone, in all cases appear as a round or ovoid hypointense region that replaces the intermediate or high signal intensity tumor seen on the preablation image. The hypointense thermal ablation zone is surrounded by a bright rim with a well-defined outer border. Thin rim enhancement is noted in all contrast-enhanced MR images [36].

Followup MR imaging must be performed every 3 months during the first year after ablation and every 6 months thereafter.

Tumor recurrence is defined as the appearance of hyperintense soft-tissue signal within the ablation zone or along its margin on T2-weighted or STIR MR images or as areas of abnormal contrast enhancement within the treated region on the postcontrast images [37].

## 5. FOLLOWUP CRYOSURGYCAL ABLATION OF SRM

Concomitantly with the change in presentation of renal masses there is a paradigm shift in the management of localized small renal lesions. Minimally invasive options such as cryoablation have emerged as an alternative surgical option for selected patients. The potential complications of nephron-sparing kidney surgery make renal cryoablation an appealing option in high-risk surgical populations.

Cryoablation requires real-time monitoring of the ice ball by ultrasound, CT, or magnetic resonance imaging (MRI), to ensure that the tumour is completely frozen and to minimize injury to the surrounding healthy tissue. However, it is preferable to use the MR imaging guidance to monitor in real time so that the entire circumference of the treatment effects can be viewed during the procedure.

The MR imaging protocol is limited to the abdomen and included: transverse T2-weighted, transverse T1-weighted sequences, and transverse fat-suppressed T1-weighted sequences before and four phases after the intravenous administration of contrast medium.

The purpose of the cryotherapy is not the excision of the tumor, but their necrosis “in situ.” The effects of renal cryoabation on the kidney have been studied in animal models [38].

The acute histologic changes are rapid coagulation necrosis and a sharp zone of transition within the normal kidney. A peripheral zone of incomplete necrosis surrounds the area of necrosis. Over time, resorption of cellular debris and fibrosis lead to shrinkage of the cryolesion.

Given that the renal lesions are treated “in situ,” a rigourous followup is required, usually with MR. Data form long-term followup examinations are crucial to asses the usefulness of cryotherapy and detecting tumor recurrence.

MR imaging and the same protocol used prior to the treatment are performed also at 24–48 hours after treatment, for assessment of complications (bleeding or urinoma).

Remer and coworkers [39] reported several characteristic findings in serial MR scans performed on the first day, 1 month, 3 months, 6 months, and 12 months after renal cryoablation. MR images are also compared with the pretreatment MR images, to determine the amount of cryonecrosis, defined as tissue that no longer appeared to be enhanced by intravenous contrast material.

The signal intensities of cryolesions on T1-and T2-weighted images were somewhat variable. Lesions were generally isointense on T1-weighted images and iso-or hypointense on T2. The borders of cryolesions were well depicted on T2-weighted images because of the relative hypointensity of the lesion compared with normal renal parenchyma.

In patients without evidence of tumor recurrence, all cryolesions showed a dramatic progressive decrease in size over time (63% and 94% at 1 month and 1 year, resp.). Some cryolesions had a peripheral hypervascularized rim on T1-weighted gadolinium enhanced images. This was seen in up to 50% of lesions imaged within the first 3 months after ablation, but was present in just 10% of lesions at 12 months. Initial rim enhancement has been reported in liver ablation cases and has been attributed to the inflammatory response.

Any increase in size of a cryolesion should be viewed with suspicion.

Although MR is the most studied method of monitoring cryolesions, CT has also been evaluated [40]. The cryolesions on followup CT showed no evidence of enhancement and the tumor demonstrate stable size or decease in size.

## APPENDIX

### THE BOSNIAK RENAL CYST CLASSIFICATION SYSTEM

Category I A benign simple cyst with a hairline-thin wall that does not contain septa, calcifications, or solid components: it measures water density and does not enhance with contrast material.

Category II A benign cyst that may contain a few hairline-thin septa: fine calcification or a short segment of slightly thickened calcification may be present in the wall or septa. Uniformly high-attenuation lesions (<3 cm) that are sharply marginated and do not enhance are included in this group.

Category IIF These cysts may contain an increased number of hairline-thin septa. Minimal enhancement of a hairline-thin smooth septum or wall can be seen, and there may be minimal thickening of the septa or wall. The cyst may contain calcification that may be thick and nodular, but no contrast enhancement is present. There are no enhancing soft-tissue components. Totally intrarenal nonenhancing high-attenuation renal lesions that are 3 cm or larger are also included in this category. These lesions are generally well marginated.

Category III These lesions are indeterminate cystic masses that have thickened irregular walls or septa in which enhancement can be seen.

Category IV These lesions are clearly malignant cystic masses that not only have all the characteristics of category III lesions, but also contain enhancing soft-tissue components adjacent to but independent of the wall or septa.
